# Prematurity and Multidimensional Risk Patterns in Adolescent and Adult Pregnancies: A Principal Component Analysis in an Eastern European Cohort

**DOI:** 10.3390/children12121673

**Published:** 2025-12-09

**Authors:** Florin Tovirnac, Alina Mihaela Calin, Catalin Plesea-Condratovici, Monica-Laura Zlati, Nicoleta Andreea Tovirnac

**Affiliations:** 1 Clinic Surgical Department, Dunarea de Jos University of Galati, 800008 Galati, Romania; tovarnacf@yahoo.com (F.T.); alina.calin@ugal.ro (A.M.C.); andreeatovarnac@yahoo.com (N.A.T.); 2 Morphological and Functional Sciences Department, Faculty of Medicine and Pharmacy, “Dunarea de Jos” University, 800008 Galati, Romania; 3 Department of Business Administration, Dunarea de Jos University of Galati, 800008 Galati, Romania

**Keywords:** preterm birth, principal component analysis, pregnancy outcomes, adolescent pregnancy, clinical risk stratification

## Abstract

**Highlights:**

**What are the main findings?**

- Principal component analysis revealed three distinct latent risk dimensions, namely—maternal–clinical, fetal–placental, and exogenous–socio-demographic, —across adolescent preterm, adult preterm and adult term pregnancies.
- The structure and strength of these risk dimensions differ by age group and pregnancy outcome, with adolescents showing the most concentrated clustering of clinical and behavioural vulnerabilities.

**What are the implications of the main findings?**

- The multidimensional and age-dependent configuration of prematurity risk supports the development of cohort-specific preventive strategies, especially targeted interventions for vulnerable adolescent pregnancies.
- The PCA-derived latent dimensions offer an empirical framework that can guide future risk-score development and clinical decision-support tools, pending validation in larger prospective and multicentre studies.

**Abstract:**

Background: Preterm birth remains a major cause of neonatal morbidity and mortality, with risk shaped by interacting maternal, fetal, placental and behavioural factors. This study examined latent multidimensional risk patterns in adolescent and adult pregnancies in an Eastern European cohort. Methods: We conducted a retrospective observational study including all non-COVID pregnant women who delivered at the County Emergency Clinical Hospital of Brăila, Romania, between 2020 and 2021. Three cohorts were analyzed: adolescent preterm mothers (Lot E; n = 54), adult preterm mothers (Lot P; n = 231) and adult term mothers (Lot M; n = 3354). Maternal, fetal, placental and behavioural indicators were coded as ordered clinical risk categories, and separate principal component analyses (PCA) with Varimax rotation were performed within each cohort. Results: Across all three groups, PCA identified three latent dimensions that together explained approximately 66–72% of the total variance. The composition of these components differed by cohort: in adolescents, maternal complications, exogenous behaviours and obstetric–placental indicators tended to cluster; in adult preterm pregnancies, placental–obstetric and behavioural indicators formed distinct but interrelated dimensions; and in adult term pregnancies, behavioural and socio-environmental indicators were the most prominent contributors to the latent structure, with fetal outcomes forming a separate dimension. Conclusions: Prematurity-related risk profiles were multidimensional and varied meaningfully by age and pregnancy outcome. These exploratory PCA-derived dimensions provide a data-driven framework for understanding how risk clusters across different maternal populations and may help generate hypotheses for age-specific preventive and clinical strategies. Confirmation and further validation in prospective, multicentre studies are required before clinical application.

## 1. Introduction

Prematurity is defined, in accordance with the World Health Organization (WHO), as birth between 22+0 and 36+6 weeks of gestation and remains a leading cause of neonatal morbidity and mortality worldwide, with little reduction in global rates over the past decade [1,2,3]. Beyond immediate neonatal complications, preterm birth is associated with long-term neurodevelopmental, metabolic and cardiovascular sequelae, generating a substantial burden for families and health systems [4,5,6,7].

The etiology of prematurity is multifactorial, involving interacting maternal clinical conditions, fetal and placental pathology, behavioural risk factors, socio-economic disadvantage and barriers in access to high-quality antenatal care [8,9,10,11]. These determinants tend to cluster within individuals and communities, generating complex multidimensional risk profiles rather than isolated single risk factors [11,12,13,14,15].

The COVID-19 pandemic further disrupted antenatal and perinatal services and amplified psychosocial stressors for pregnant women; however, population-level trends in preterm birth during this period were heterogeneous, reflecting differences in baseline risk, health-care organization and health-seeking behaviours across settings [16,17,18,19,20,21].

Romania has one of the highest prematurity rates in the European Union and a persistently elevated burden of adolescent pregnancy [22,23]. Vulnerable groups (including teenage mothers, women from socio-economically deprived or rural communities and those with limited access to specialized obstetric care) are disproportionately exposed to nutritional deficits, anemia, infections, smoking, alcohol and other health-risk behaviours, as well as systemic barriers within the maternity care system [24,25,26,27,28,29]. These factors often cluster within the same individuals and communities, leading to complex multidimensional risk profiles rather than isolated single risk factors.

Traditional epidemiological studies have typically examined maternal, fetal, placental and exogenous determinants of prematurity separately, providing a segmented view of risk [30,31,32,33,34,35]. In contrast, multivariate dimension-reduction techniques such as principal component analysis (PCA) can summarize patterns of co-variation across multiple risk indicators, revealing latent risk dimensions that may differ between subgroups of pregnant women [36,37,38,39].

Adolescent mothers differ from adult mothers with respect to biological maturation, socio-demographic profile and behavioural risk exposure, and adult women who deliver preterm may also have risk structures distinct from those who deliver at term. We therefore hypothesized that the latent organization of prematurity-related risk factors would vary across adolescent preterm pregnancies, adult preterm pregnancies and adult term pregnancies. Drawing upon a representative cohort of non-COVID pregnant patients delivering in a tertiary maternity hospital in eastern Romania, we used PCA to characterize multidimensional risk patterns in these three groups, with the aim of informing more targeted clinical and public health strategies to prevent preterm birth.

## 2. Materials and Methods

### 2.1. Study Design

This study employed a retrospective observational design based on routinely collected medical records from the County Emergency Clinical Hospital of Brăila, Romania (Unit D), covering the period of 2020–2021. The institution functions as a tertiary maternity centre and provides obstetric and neonatal care for both urban and rural populations in Brăila County and the surrounding regions.

### 2.2. Participants

All deliveries registered in Unit D during the study period were screened for eligibility. We included pregnant women who delivered at the study hospital between 2020 and 2021, had a documented gestational age, and presented complete data for the maternal, fetal, placental and exogenous risk indicators listed in Table 1. Pregnancies with confirmed SARS-CoV-2 infection at the time of delivery were excluded, as were cases with incomplete information on the core variables required for the multivariate analyses, to ensure comparability across the analytic groups.

To account for the marked heterogeneity associated with maternal age and gestational maturity—two dimensions known to influence both the distribution and clustering of clinical risk factors—the eligible women were classified into three analytically distinct cohorts: an adolescent preterm group (Lot E; maternal age < 20 years and preterm delivery), an adult preterm group (Lot P; maternal age ≥ 20 years and preterm delivery), and an adult term reference group (Lot M; maternal age ≥ 20 years and term delivery). The cohort sizes were as follows: Lot E, 54 cases; Lot P, 231 cases; Lot M, 3354 cases.

This classification allowed the subsequent multivariate analyses (including PCA) to be conducted within clinically homogeneous groups, thereby ensuring that any latent structures identified in the risk profiles reflect genuine internal patterns of association rather than confounding influences driven by age-related or gestational differences. Lot E was considered the primary vulnerable group; Lot P served as the adult preterm comparison group; and Lot M acted as the term-birth reference cohort.

### 2.3. Measures

Data were extracted from obstetric, neonatal and laboratory records by trained clinicians using a structured data collection form. The variables of interest covered four clinical domains: maternal risk indicators, fetal and neonatal indicators, placental indicators and exogenous (behavioural and socio-educational) indicators. Each indicator was defined as an ordered clinical risk category, reflecting a graded increase in severity or unfavourable prognosis, as detailed in Table 1.

All variables followed a clinically meaningful ordinal structure, with higher integer codes corresponding to higher levels of maternal, fetal, placental or exogenous risk. This approach ensured internal consistency across domains and allowed the ordered risk scores to be used both in descriptive analyses and as input variables for the principal component analyses (PCA). The use of ordinal clinical risk indicators in PCA is consistent with established practice in perinatal epidemiology, as the monotonic risk gradients embedded in these categories support the identification of latent structures across heterogeneous risk dimensions.

### 2.4. Data Analysis

All data were processed and analyzed using IBM SPSS Statistics for Windows version 27 (IBM Corp., Armonk, NY, USA) [40]. Two-sided *p*-values < 0.05 were considered statistically significant. Given the ordered structure of the clinical risk indicators, principal component analysis (PCA) was subsequently applied within each cohort to identify latent patterns of association among maternal, fetal, placental and exogenous risk factors. The analytic procedures and criteria for component extraction are detailed in the corresponding section.

#### 2.4.1. Descriptive Statistics and Initial Comparisons

Descriptive statistics were computed for all variables. Continuous variables were reported as mean ± standard deviation (SD) or median with interquartile range (IQR), as appropriate. All categorical clinical risk indicators were summarized using absolute frequencies (N) and proportions (%). A complete distribution of these ordered risk categories across Lots E, P and M is provided in Appendix A. The observed patterns highlight the accumulation of unfavourable maternal, fetal, placental and exogenous conditions among preterm pregnancies, thereby offering essential empirical context for the subsequent PCA models and supporting the identification of latent structures within each cohort.

#### 2.4.2. Correlation Analyses

Because many of the clinical risk indicators were ordinal and exhibited non-normal distributions, bivariate associations among them were examined using Spearman’s rank correlation coefficients (rho). Complete correlation matrices for all variables, were computed separately for each cohort. Inspection of these matrices revealed several coherent and clinically meaningful patterns. Strong positive associations were observed among the exogenous risk indicators (ER_Alcohol, ER_Smoking and ER_Vices), reflecting their shared behavioural and social determinants (rho up to 0.90). Likewise, fetal risk markers such as FR_TypeB and FR_SPCD showed robust correlations with placental environment indicators (e.g., PR_EnvUR), consistent with their clinical interdependence. Maternal risk indicators (MR_DM and MR_HBP) also displayed moderate positive associations (rho = 0.58), reflecting expected comorbidity patterns. The correlation structure confirmed the presence of non-redundant but interrelated risk dimensions, supporting the suitability of principal component analysis (PCA) for identifying latent patterns of risk aggregation. These correlations provided the empirical foundation for interpreting the PCA components and ensured that subsequent component loadings align with the observed bivariate relationships.

#### 2.4.3. Principal Component Analysis (PCA) for Risk Structure Identification

To identify latent multidimensional patterns among the clinical risk indicators, we performed separate principal component analyses (PCA) with orthogonal rotation for each cohort (Lots E, P and M). This analytic choice ensured that adolescent preterm pregnancies, adult preterm pregnancies and adult term pregnancies (groups that differ substantially in their biological, behavioural and sociodemographic characteristics) were not forced into a single pooled risk structure dominated by the largest cohort. Running three independent PCA models allowed us to capture cohort-specific configurations of risk that would otherwise have been obscured.

Although the indicators were originally coded as categorical or ordinal variables, they represent ordered gradations along underlying continuous clinical constructs (e.g., placental dysfunction, fetal compromise, maternal metabolic burden, intensity of exogenous risk). Consistent with established practices in perinatal epidemiology, these ordered categories were treated as monotonic risk scores, and PCA was applied to the correlation matrix of the standardized indicators. This approach is commonly used when the aim is dimensionality reduction and extraction of clinically interpretable latent structures. Alternative methods, such as categorical PCA or multiple correspondence analysis, were considered but not adopted to preserve transparency and comparability with prior literature; this is acknowledged as a methodological limitation. The suitability of the data for PCA was confirmed using the Kaiser–Meyer–Olkin (KMO) measure and Bartlett’s test of sphericity, with KMO values ≥ 0.60 and significant Bartlett tests (*p* < 0.05) indicating adequate sampling adequacy. The Spearman correlation matrices for each cohort, revealed coherent, clinically meaningful, and distinctly patterned associations among the risk indicators. These cohort-specific correlation structures justified both the use of PCA and the extraction of separate components for each group, ensuring that the interpretation of components is fully aligned with the observed data patterns.

Components were extracted using eigenvalues > 1.0, supported by scree plot inspection and clinical interpretability criteria. An orthogonal Varimax rotation was applied to enhance simple structure and improve interpretability. A loading threshold of |0.40| was used to identify salient variables within each component. Component interpretation was based strictly on (i) the pattern and magnitude of loadings and (ii) the correlation structures, ensuring full consistency between empirical data and conceptual labelling. Factor scores were subsequently computed for each retained component and used in secondary comparisons across cohorts.

## 3. Results

This section reports the empirical results derived from the analysis of the collected clinical and socio-demographic data. We first present the descriptive distributions of the maternal, fetal, placental and exogenous risk indicators across the three cohorts. These descriptive findings are complemented by the cohort-specific Spearman correlation matrices, which document the underlying patterns of association among the ordered clinical risk variables. Together, these preliminary results provide the empirical foundation for interpreting the latent structures identified through the subsequent Principal Component Analysis (PCA). The PCA findings are then presented for each cohort separately, reflecting the decision to estimate distinct models for adolescent preterm pregnancies (Lot E), adult preterm pregnancies (Lot P) and adult term pregnancies (Lot M). This approach allows the latent components to be interpreted in direct alignment with the cohort-specific covariance patterns observed in the correlation analyses, ensuring conceptual and empirical consistency throughout the results.

### 3.1. Descriptive Statistics

Descriptive statistics of the three cohorts are summarized in Table 2.

The adolescent preterm group (Lot E, 54 cases) and the adult preterm group (Lot P, 231 cases) were much smaller than the adult term reference group (Lot M, 3354 cases). Mean ordered risk scores for prematurity severity (PR_PREMATURE) and adverse fetal outcomes (FR_BMALF and FR_SPCD) were higher in Lots E and P than in Lot M, indicating a greater burden of unfavourable fetal conditions among preterm pregnancies. Exogenous behavioural risks (ER_Smoking, ER_Alcohol and ER_Vices) and maternal comorbidities (MR_DM and MR_HBP) also had higher mean risk scores in Lots E and P compared with Lot M, while placental risks related to residence and mode of delivery (PR_EnvUR and PR_TypeB) tended to be less favourable in the preterm groups. Overall, these descriptive findings suggest more concentrated and multidimensional risk profiles among adolescent and adult preterm pregnancies than among adult term pregnancies. They support the use of principal component analysis (PCA) to summarize the joint variation in the selected maternal, fetal, placental and exogenous risk indicators.

### 3.2. Correlation Analysis

The correlation analysis summarized in Table 3 reveals coherent and clinically meaningful associations among the maternal, fetal, placental and exogenous risk indicators across the three cohorts.

The cohort-specific structure of these associations confirms that the risk indicators do not operate independently but cluster along meaningful clinical dimensions. These correlation matrices provided the empirical groundwork for the principal component analyses by identifying the covariance patterns that underlie the latent risk structures. The PCA component interpretations presented in the subsequent sections were derived directly from these observed associations, ensuring full consistency between the empirical data and the conceptual labelling of the components.

#### 3.2.1. Placental Risk: Residence and Birth Type

Across all three cohorts, PR_EnvUR (urban vs. rural residence) exhibited strong and statistically significant negative correlations with FR_TypeB (mode of birth), with Spearman coefficients ranging from −0.74 to −0.90 (Lot E: ρ = −0.741; Lot P: ρ = −0.896; Lot M: ρ = −0.744; all *p* < 0.001). These values indicate a stable pattern in which urban residence is associated with higher FR_TypeB scores—reflecting a greater likelihood of cesarean delivery—whereas rural residence is more often associated with vaginal birth. The association was strongest in the adult preterm cohort (Lot P), suggesting that, among preterm adult pregnancies, contextual factors linked to living environment may have a particularly pronounced relationship with delivery mode. Taken together, these correlations delineate a consistent placental-obstetric risk dimension across the three groups. They also provide direct empirical support for the subsequent PCA, in which PR_EnvUR and FR_TypeB systematically co-loaded on the same latent component, reflecting their shared covariance structure.

#### 3.2.2. Exogenous Risk and Knowledge Level

Across the adult cohorts, the association between educational attainment (PR_Studies) and smoking risk (ER_Smoking) showed divergent patterns. In the adult preterm group (Lot P), there was a strong positive correlation (ρ = 0.645, *p* < 0.001), indicating that higher smoking risk scores tended to co-occur with less favourable educational profiles. By contrast, in the adult term group (Lot M), the correlation was very small (ρ = 0.033, *p* = 0.03) and, despite reaching statistical significance due to the large sample size, is of negligible clinical relevance. In the adolescent cohort (Lot E), PR_Studies and ER_Smoking were modestly and inversely related (ρ = −0.432, *p* = 0.001), suggesting that lower levels of education were more frequently associated with higher smoking risk among pregnant adolescents. Taken together, these cohort-specific patterns point to age- and context-dependent links between educational disadvantage and exogenous risk behaviours. They also support the PCA results, in which smoking, alcohol use and related exogenous indicators cluster with educational factors in distinct ways across the three cohorts, reflecting the underlying covariance structure documented in Table 3.

#### 3.2.3. Prematurity Severity and Fetal Outcomes

In both preterm cohorts, higher prematurity severity (PR_PREMATURE) was strongly associated with more adverse neonatal discharge outcomes (FR_SPCD). The correlation (Table 3) was very strong in adolescents (Lot E: ρ = 0.876, *p* < 0.001) and moderate among adult preterm pregnancies (Lot P: ρ = 0.489, *p* < 0.001). This pattern indicates that increasing degrees of prematurity are consistently linked to more severe neonatal compromise, with a notably steeper gradient in the adolescent group. This association could not be meaningfully assessed in the adult term cohort (Lot M), where prematurity severity is invariant. The cohort-specific gradients observed in Lots E and P provided an essential empirical anchor for the PCA structure, where prematurity severity and neonatal outcomes contributed jointly to a shared latent risk dimension.

#### 3.2.4. Maternal Comorbidities and Associated Risks

In the adolescent preterm cohort (Lot E), maternal hypertension (MR_HBP) showed a moderate and statistically significant positive correlation with prematurity severity (PR_PREMATURE) (ρ = 0.502, *p* < 0.001), indicating that hypertensive disorders tended to co-occur with more severe degrees of prematurity. In contrast, this association was absent in the adult preterm cohort (Lot P: ρ = −0.065, *p* = 0.164), suggesting that hypertension was not a distinguishing correlate of prematurity severity among adult mothers. As expected, the association could not be meaningfully evaluated in the term cohort (Lot M), where prematurity severity is invariant. Alcohol consumption (ER_Alcohol) also demonstrated age-dependent correlation patterns with neonatal discharge outcomes (FR_SPCD). Among adolescents, higher alcohol risk scores were positively associated with more adverse discharge outcomes (ρ = 0.339, *p* = 0.006), whereas in adult preterm pregnancies the relationship was inverse (Lot P: ρ = −0.312, *p* < 0.001). These divergent associations point to cohort-specific configurations of maternal comorbidities and exogenous risk behaviours in relation to prematurity and early neonatal outcomes.

Collectively, these patterns reinforce the rationale for analyzing adolescent and adult pregnancies separately. They also align with the PCA findings, where maternal comorbidity indicators and exogenous behaviours contributed differentially to latent components across cohorts, reflecting the underlying covariance structures observed in Table 3.

### 3.3. Principal Component Analysis (PCA) Suitability and Extraction

Building on the cohort-specific correlation patterns described above, we first assessed the suitability of the clinical risk indicators for principal component analysis (PCA). The Spearman correlation matrices demonstrated coherent clusters of moderate-to-strong associations among maternal, fetal, placental and exogenous risk variables, indicating that the dataset satisfied the fundamental requirement for a shared underlying covariance structure. This preliminary evidence, together with acceptable values for the Kaiser–Meyer–Olkin (KMO) statistic and significant Bartlett tests of sphericity in each cohort, confirmed that the data were appropriate for dimensionality reduction.

Following this verification, PCA was performed separately in each cohort. Component extraction was based on the eigenvalue > 1.0 criterion, supported by scree plot inspection and the clinical interpretability of the rotated loading patterns. Varimax rotation was applied to achieve a clearer simple structure. The resulting components represent cohort-specific latent risk dimensions, directly aligned with the empirical associations observed in the correlation analyze.

#### 3.3.1. Kaiser-Meyer-Olkin (KMO) Measure of Sampling Adequacy and Bartlett’s Test of Sphericity

Table 4 summarizes the Kaiser–Meyer–Olkin (KMO) measure of sampling adequacy and Bartlett’s test of sphericity for the three cohorts.

The KMO values were 0.609 for adolescents (Lot E), 0.718 for adult preterm pregnancies (Lot P) and 0.749 for adult term pregnancies (Lot M), indicating acceptable sampling adequacy in Lot E and good adequacy in both adult cohorts. Bartlett’s tests were highly significant in all groups (Lot E: χ^2^ = 521.465, df = 66; Lot P: χ^2^ = 1723.008, df = 66; Lot M: χ^2^ = 21,126.927, df = 55; all *p* < 0.001), confirming that the correlation matrices departed sufficiently from identity and were therefore appropriate for factor extraction. Together with the structured patterns of associations observed in the Spearman matrices, these diagnostics support the suitability of applying PCA in each cohort and provide a robust statistical foundation for extracting cohort-specific latent risk dimensions.

#### 3.3.2. Communality Values and Variable Retention

Table 5 reports the communalities for each risk indicator within each cohort, representing the proportion of each variable’s variance accounted for by the retained components.

In the adolescent preterm cohort (Lot E), most indicators displayed relatively high communalities, generally above 0.60. The largest values were observed for prematurity severity (PR_PREMATURE; 0.883), maternal hypertension (MR_HBP; 0.867) and placental risk associated with residential environment (PR_EnvUR; 0.807). These results indicate that these variables contributed strongly to the shared variance underlying the latent risk structure specific to adolescent pregnancies.

In the adult preterm cohort (Lot P), communalities were more moderate overall, yet key variables continued to show satisfactory variance extraction. High communalities were found for mode of birth (FR_TypeB; 0.836), alcohol-related exogenous risk (ER_Alcohol; 0.845), and behavioural risk clustering (ER_Vices; 0.816). Prematurity severity (PR_PREMATURE; 0.701) also showed substantial shared variance. Conversely, maternal hypertension (MR_HBP; 0.367) contributed only modestly to the common factor space, suggesting that this indicator was less integrated into the multidimensional risk pattern among adult preterm pregnancies compared with adolescents.

In the adult term cohort (Lot M), communalities ranged from moderate to high (approximately 0.44–0.82). The indicators with the strongest contributions were PR_EnvUR (0.808), FR_TypeB (0.777) and ER_Smoking (0.756), signalling that placental environment, delivery mode and exogenous behavioural risks represented core shared dimensions in this cohort. Educational attainment (PR_Studies; 0.438) showed the lowest communality, indicating a weaker alignment with the latent risk structure in term pregnancies. As expected, PR_PREMATURE was not included in Lot M due to absence of variability.

Overall, the communality values across the three cohorts support the adequacy of the selected indicators for PCA and highlight meaningful differences in how maternal, fetal, placental and exogenous risks contribute to the underlying multidimensional structure of adolescent, adult preterm and adult term pregnancies.

#### 3.3.3. Eigenvalues and Variance Explained

Table 6 presents eigenvalues and the proportion of variance explained by each component for the three PCA models.

In all cohorts, three components had eigenvalues greater than 1.0 and were therefore retained as the final component structure (Table 6). In the adolescent preterm cohort (Lot E), the first component accounted for 41% of the total variance, and the first three components together explained approximately 71.9%. In adult preterm pregnancies (Lot P), the first component explained 37.8% of the variance, with the first three components cumulatively accounting for 66.1%. A similar pattern was observed among adult term pregnancies (Lot M), where the first component explained 37.6% and the first three components together explained 66.9% of the total variance.

**Figure 1:**
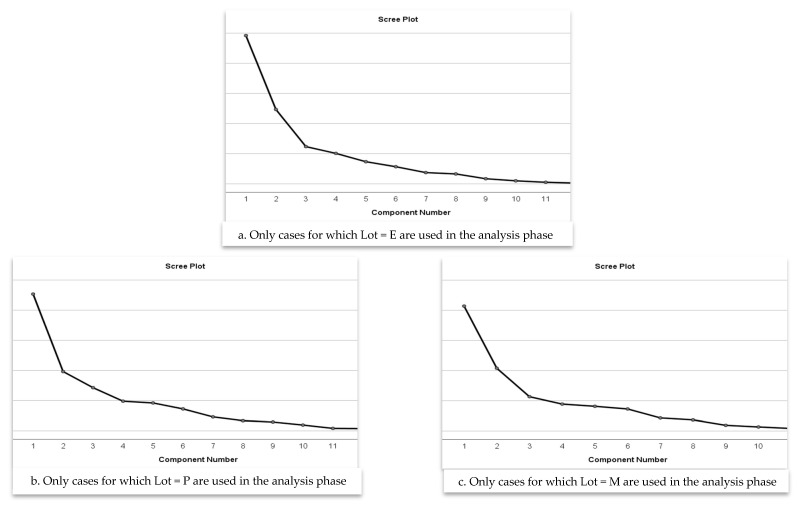
Scree Plot for determining the optimal number of principal components in the PCA analysis, shown separately for Lots E, P and M.

These results indicate that, in each cohort, a three-component solution captured a substantial proportion of the information contained in the original indicators, supporting the adequacy of dimensionality reduction. After Varimax rotation, the variance was distributed more evenly across components—without altering the total variance explained—thereby enhancing interpretability and facilitating the identification of coherent latent risk dimensions within each cohort. The scree plots (Figure 1) further supported the retention of three components in all groups, as each plot displayed a clear inflection after the third component.

### 3.4. Identification and Interpretation of Latent Risk Dimensions

This subsection summarizes the factor loading patterns that define the latent risk dimensions identified by PCA in each cohort.

#### 3.4.1. Factor Loading Matrix—Cohort-Specific Interpretation

Table 7 presents the component loading matrix obtained from the PCA models in the three cohorts and shows the contribution of each risk indicator to the retained components.

In Lot E, the first component was characterized by high positive loadings for maternal hypertension (MR_HBP; 0.896), placental environment (PR_EnvUR; −0.819), mode of birth (FR_TypeB; 0.819), exogenous risks such as alcohol use (ER_Alcohol; 0.763), and prematurity severity (PR_PREMATURE; 0.677). This pattern indicates a combined maternal–placental–obstetric–behavioural risk dimension, in which adverse maternal conditions, placental vulnerability and exogenous behaviours co-occur within adolescent preterm pregnancies. The second component was defined primarily by fetal and neonatal indicators, with high loadings for FR_BMALF (0.518), FR_SPCD (0.590) and FR_THRH (0.423), representing a fetal morbidity–neonatal outcome dimension. The third component included substantial contributions from educational level (PR_Studies; 0.569) and smoking behaviours (ER_Smoking; −0.657), reflecting a socio-behavioural gradient that is distinct from the medical risk dimensions.

In adult preterm pregnancies (Lot P), the first component showed dominant loadings for PR_EnvUR (−0.832), FR_TypeB (0.820), and exogenous behaviours including ER_Alcohol (0.684), ER_Smoking (0.745) and ER_Vices (0.633). Prematurity severity (PR_PREMATURE; −0.549) also contributed moderately. This configuration reflects a broad placental-obstetric-behavioural risk dimension, consistent with the strong correlations observed between environmental, obstetric and lifestyle indicators in this cohort. The second component was defined mainly by neonatal conditions, with high loadings for FR_BMALF (0.658) and FR_SPCD (0.376), indicating a neonatal morbidity factor. The third component showed salient positive loading for maternal diabetes (MR_DM; 0.746), suggesting a maternal metabolic dimension that is less prominent than in adolescents but nonetheless distinct within the adult preterm profile.

In the adult term cohort (Lot M), the first component was dominated by exogenous indicators—smoking (ER_Smoking; 0.856), alcohol use (ER_Alcohol; 0.840), and cumulative vices (ER_Vices; 0.811)—together with placental environment (PR_EnvUR; –0.853). This configuration represents a pronounced exogenous–socio-environmental risk dimension, consistent with the strong behavioural clustering seen in the correlation matrix. The second component was defined by fetal and neonatal risks, including FR_SPCD (0.675), FR_THRH (0.544) and FR_BMALF (0.436), forming a fetal–neonatal morbidity dimension. The third component included contributions from maternal diabetes (MR_DM; −0.273) and mode of birth (FR_TypeB; 0.185), representing a more modest maternal–obstetric dimension relative to adolescents and preterm adults.

#### 3.4.2. Interpretation of Rotated Components

Table 8 presents the correlations between the initial and the Varimax-rotated components for each cohort.

In all three groups, each rotated component showed a strong correlation with one of the initial components, indicating that the Varimax rotation preserved the substantive variance structure while redistributing the loadings to achieve a clearer simple structure. This pattern confirms that the rotation did not alter the underlying dimensionality of the data but improved the separation of the latent factors.

Importantly, the transformation matrices should be interpreted solely as indicators of component stability and orthogonality rather than as clinical descriptors. The values demonstrate that the rotated solutions remained mathematically consistent with the original component space, with each rotated component corresponding predominantly to a single initial axis of variation. This coherence supports the reliability of the rotated loading patterns, which form the basis for the clinical interpretation of latent dimensions.

The transformation matrix therefore validates the use of Varimax rotation as a means of enhancing interpretability without modifying the total variance explained or the substantive content of the extracted components. It confirms that the rotated solutions provide a stable and conceptually coherent representation of the covariance structure within each cohort, allowing the multidimensional risk patterns to be interpreted with greater clarity and without implying causal relationships among the underlying factors.

**Figure 2:**
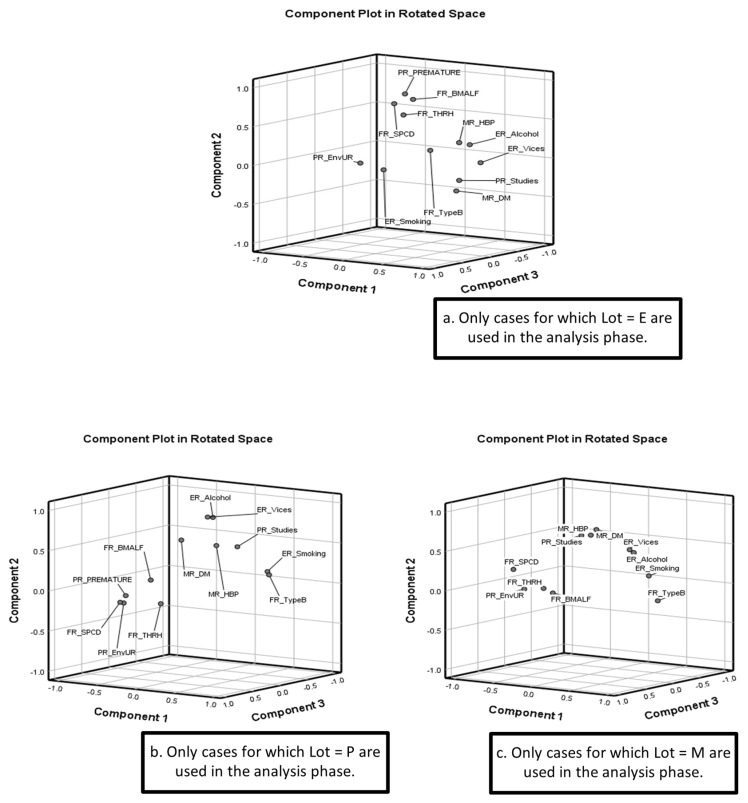
Three-dimensional representation of the principal components in rotated space for the analyzed batches: (**a**) Lot E, (**b**) Lot P and (**c**) Lot M.

Figure 2 illustrates the three-dimensional distribution of the risk indicators within the rotated PCA space for each cohort (Lots E, P and M), providing a visual complement to the factor loadings presented in Table 7. The plots show how variables group together according to their shared variance and help clarify the latent dimensions identified in the previous subsection.

In Lot E (adolescent preterm pregnancies), indicators that were strongly associated in the correlation matrix—such as PR_EnvUR, FR_TypeB, and exogenous risks (ER_Smoking, ER_Alcohol, ER_Vices)—cluster in close proximity within the PCA space, reflecting their high cross-loadings on Component 1. Variables related to prematurity and fetal outcomes (PR_PREMATURE, FR_SPCD, FR_BMALF) appear closer together along Components 2 and 3, consistent with the moderate to strong correlations observed in this cohort and with their shared loadings on the fetal-outcome dimension of the PCA. Maternal comorbidities (MR_HBP, MR_DM) project in positions that reflect their more modest but still meaningful communalities, indicating partial integration within the broader risk structure of adolescent pregnancies.

In the adult preterm cohort (Lot P), Figure 2 shows a clearer separation between exogenous risks and clinical–obstetric indicators. Exogenous behaviours (ER_Smoking, ER_Alcohol, ER_Vices) cluster closely, consistent with their high inter-correlations and their strong loadings on Component 1. Obstetric and placental variables (FR_TypeB, PR_EnvUR) occupy a distinct region of the component space, reflecting the coherent placento-obstetric dimension identified in the PCA. Maternal comorbidities (MR_HBP, MR_DM) show more dispersed positions, consistent with their lower communalities and weaker associations with the other indicators in Lot P.

In the adult term cohort (Lot M), the component space displays a more heterogeneous arrangement of variables. Exogenous risks again appear grouped, reflecting the strong associations among smoking, alcohol consumption and cumulative vices. Placental and obstetric factors (PR_EnvUR, FR_TypeB) are projected in close proximity, in line with their high shared variance in Table 7 and with the patterns seen in the correlation matrix. Maternal risk indicators (MR_DM, MR_HBP) appear more diffusely positioned, which is consistent with the more modest correlations and lower communalities observed in Lot M. Fetal indicators (FR_BMALF, FR_THRH, FR_SPCD) occupy positions reflecting their moderate cross-loadings on the fetal-clinical dimension.

Across all three cohorts, Figure 2 visually reinforces the factor-loading patterns reported in the PCA: a placento-obstetric dimension dominated by PR_EnvUR and FR_TypeB; an exogenous-behavioural dimension represented by ER_Smoking, ER_Alcohol and ER_Vices; and a fetal-outcome dimension, more prominent in the preterm cohorts, clustering PR_PREMATURE with FR_SPCD and other fetal indicators.

These visual clusters support the multidimensional risk structure identified through PCA and highlight consistent patterns across age-defined cohorts without suggesting causal determinants.

### 3.5. Comparative Analysis of Principal Component Scores

Table 9 reports the component score coefficient matrices for all three cohorts, representing the weights used to compute individual scores on each rotated principal component. These coefficients should be interpreted as the contribution of each standardized indicator to the computation of component scores, and not as direct factor loadings. Their pattern, when examined alongside the loading matrices presented earlier, provides insight into how variables combine to define the main latent dimensions in each group.

In the adolescent cohort (Lot E), the coefficients for Component 1 include positive contributions from MR_DM (0.225), MR_HBP (0.208), ER_Alcohol (0.227) and ER_Vices (0.266), together with a negative weight for PR_EnvUR (−0.192). This configuration reflects the multidimensional nature of the first latent dimension in Lot E, in which maternal comorbidities, exogenous behaviours and residential context jointly contribute to the component score, consistent with the high communalities and strong cross-loadings observed in Table 7. Component 2 shows high positive contributions from PR_PREMATURE (0.330), FR_BMALF (0.335) and FR_SPCD (0.267), reflecting the fetal and neonatal clinical dimension identified earlier in the PCA. These positive weights indicate that higher scores on this component correspond to pregnancies marked by more severe prematurity and more adverse neonatal outcomes. Component 3 is characterized by the strongest positive coefficient for ER_Smoking (0.521) and a substantial negative coefficient for PR_Studies (−0.489), indicating a distinct behavioural–socioeducational gradient, separate from the clinical dimensions represented in Components 1 and 2.

In the adult preterm cohort (Lot P), Component 1 combines contributions from FR_TypeB (0.322), MR_DM (−0.334) and PR_EnvUR (−0.319). This pattern mirrors the structure of Component 1 in the loading matrix, reflecting a dimension that integrates obstetric mode of birth with maternal metabolic status and residential environment. Component 2 is dominated by exogenous indicators, including ER_Vices (0.382), ER_Alcohol (0.371) and ER_Smoking (0.195), reflecting a behavioural lifestyle component that is distinct in Lot P. This is consistent with the strong clustering of these variables in the PCA plot and their moderate-to-high communalities. Component 3 shows notable contributions from PR_PREMATURE (0.348) and FR_BMALF (0.360), consistent with the prematurity–fetal outcome dimension identified earlier. The presence of fetal indicators in this component parallels the second and third components observed in adolescent pregnancies, though with cohort-specific variation.

In the adult term cohort (Lot M), Component 1 retains substantial positive coefficients for ER_Smoking (0.223), ER_Alcohol (0.138) and ER_Vices (0.118), reflecting a behavioural–exogenous dimension similar in structure to that observed in the preterm adults. Placental environment (PR_EnvUR; −0.255) also contributes to this dimension, indicating an intertwined socio-environmental component. Component 2 shows notable weights for PR_Studies (0.397) and MR_HBP (0.336), suggesting that this dimension reflects a maternal–socio-demographic axis where educational level and hypertensive conditions contribute jointly to the score, consistent with their moderate communalities in Table 7. Component 3 includes large positive coefficients for FR_BMALF (0.546) and FR_THRH (0.575), identifying a fetal outcome dimension in Lot M. These high coefficients are aligned with the loading structure and confirm that fetal indicators form a distinct latent component in the term cohort.

Across all three groups, the component score coefficients reinforce the multidimensional nature of the underlying risk structure: Lot E: predominantly integrates maternal–behavioural factors (Comp. 1), fetal–prematurity severity (Comp. 2), and a distinct behavioural–educational axis (Comp. 3); Lot P: shows a placento–obstetric–metabolic dimension (Comp. 1), a clear behavioural component (Comp. 2), and a prematurity–fetal outcome component (Comp. 3); Lot M: separates into a behavioural–environmental component (Comp. 1), a maternal–socio-demographic component (Comp. 2), and a fetal outcome component (Comp. 3).

These results confirm that the PCA components capture cohort-specific combinations of maternal, fetal, placental and behavioural indicators, and that the underlying latent structures vary in meaningful ways across adolescent, adult preterm and adult term pregnancies.

## 4. Discussion

### 4.1. Validation of Multidimensional Risk Structure

The principal component analyses conducted across the three cohorts yielded stable and statistically well-supported latent structures. In all groups, sampling adequacy was acceptable to good, as indicated by KMO values ranging from 0.609 to 0.749, while Bartlett’s tests were highly significant, confirming that the correlation matrices were suitable for factor extraction. Communality estimates were generally satisfactory, with most indicators contributing meaningfully to the shared variance, and three components with eigenvalues greater than 1.0 collectively accounted for approximately two-thirds of the total variance. These diagnostics indicate that a relatively compact set of latent dimensions captured the core patterns of co-variation among the maternal, placental, fetal and behavioural risk indicators [41,42,43,44].

The composition and relative prominence of these components differed across the adolescent preterm, adult preterm and adult term cohorts. These variations reflect the cohort-specific correlation structures: in adolescents, maternal comorbidities and exogenous behaviours tended to co-occur with obstetric and placental indicators; in adult preterm pregnancies, placental and behavioural risks formed a distinct constellation; and in term pregnancies, behavioural indicators clustered more strongly, while fetal outcomes structured a separate dimension. Such differences emphasize that the extracted components represent empirical groupings of interrelated risk markers rather than discrete causal mechanisms. Within this framework, PCA functions as an exploratory approach that organizes complex clinical information into a reduced number of interpretable dimensions aligned with the covariance patterns observed in each cohort. By revealing how risk markers cluster differently across age and outcome categories, the PCA results provide a coherent statistical foundation for understanding the multidimensional nature of prematurity risk. These findings reinforce the view that prematurity reflects the interplay of overlapping clinical, behavioural and socio-environmental determinants, and underscore the relevance of cohort-specific, data-driven approaches to risk assessment and early intervention in obstetric care [41,42,43,45,46].

### 4.2. Comparative Interpretation of Latent Risk Profiles

The Varimax-rotated solutions enhanced the interpretability of the PCA models by concentrating high loadings on distinct components and reducing cross-loadings, thereby clarifying the multidimensional risk patterns within each cohort. When examined together, the three solutions reveal both shared and cohort-specific organizational features of maternal, fetal, placental and behavioural risks.

#### 4.2.1. Predominant Multidimensional Risk Patterns in the Adolescent Cohort (Lot E)

In the adolescent preterm cohort (Lot E), the first component integrated contributions from maternal comorbidities, exogenous behaviours and placental–obstetric indicators, reflecting a broad constellation of interrelated risks [47,48,49,50]. This clustering is consistent with the strong correlations observed between hypertension, exogenous exposures and delivery-related variables in this age group. A second component grouped fetal and neonatal indicators, capturing a coherent clinical dimension centred on prematurity severity and adverse neonatal outcomes. A third dimension reflected a socio-behavioural gradient, where smoking patterns and educational attainment contributed differentially to the latent structure. Together, these components highlight a tightly interwoven risk profile in adolescent pregnancies, in which behavioural, maternal and placental factors frequently co-occur.

#### 4.2.2. Distinct Multidimensional Risk Patterns in the Adult Preterm Cohort (Lot P)

In the adult preterm cohort (Lot P), the risk structure displayed partial overlap with adolescents but showed a more distinct separation between behavioural and clinical–obstetric indicators. Exogenous behaviours clustered strongly on one component, while placental environment and mode of birth occupied another, indicating a clearer differentiation between lifestyle-related and obstetric–clinical dimensions. Prematurity-related neonatal outcomes formed a separate component, suggesting that fetal risk patterns in adults are more isolated from maternal and behavioural contributors than in adolescents [51,52,53,54,55,56,57,58,59,60,61]. This reflects a more modular organization of risks in adult preterm pregnancies.

#### 4.2.3. Dominant Behavioural and Environmental Dimensions in the Adult Term Cohort (Lot M)

In the adult term cohort (Lot M), the latent structure was characterized by a prominent behavioural–environmental component, with smoking, alcohol use and cumulative vices forming a stable cluster alongside placental residential context [62,63,64,65,66,67,68,69,70,71]. A second component was driven by maternal and socio-demographic indicators, while fetal outcomes constituted a distinct third component. Compared with the preterm cohorts, the term risk architecture was more dispersed, consistent with lower overall clinical risk and weaker interdependencies among indicators.

Taken together, these cohort-specific profiles illustrate that although certain risk constellations (such as the linkage between exogenous behaviours and placental environment) appear consistently across groups, their relative prominence and interrelationships differ markedly by age and pregnancy outcome. This reinforces the conclusion that prematurity risk is not governed by a single dominant pathway but arises from varying combinations of behavioural, clinical and environmental factors. The PCA results therefore provide a nuanced, empirically grounded depiction of heterogeneous risk structures that can inform tailored approaches to screening and early intervention within different maternal populations.

### 4.3. Literature Context in Romania and Eastern Europe

Romania and several other Eastern European countries report some of the highest rates of adolescent pregnancy and prematurity in the European Union, in the context of persistent socio-economic inequalities and uneven access to specialized obstetric care [22,23,25,27,28,72,73,74]. Most previous studies from the region have focused on individual risk factors—such as maternal age, parity, infection or smoking—and have rarely examined how these determinants cluster within the same pregnancies.

By applying PCA to a comprehensive set of maternal, fetal, placental and exogenous indicators in adolescent and adult pregnancies, our study complements this literature with a multidimensional perspective on risk. The identification of distinct component structures in adolescent preterm, adult preterm and adult term pregnancies suggests that the same nominal risk factors may have different implications depending on the broader constellation of co-occurring risks, which is particularly relevant in settings with constrained resources and high baseline vulnerability [22,23,72,73,74].

### 4.4. Interpretation in the Context of Pandemic-Era Literature

The study period (2020–2021) coincided with the COVID-19 pandemic, which substantially reshaped antenatal care organization, health-seeking behaviours and psychosocial stress among pregnant women [73,75,76,77,78,79,80]. Although pregnancies with confirmed SARS-CoV-2 infection at delivery were excluded to avoid direct biological effects of infection on obstetric and neonatal outcomes, indirect consequences of the pandemic were likely to influence the risk patterns observed. These include reduced access to routine antenatal consultations, changes in lifestyle and mental health, altered help-seeking behaviour, and shifts in health-system resource allocation.

Our analyses cannot disentangle pandemic-related effects from pre-existing structural vulnerabilities, nor can they quantify the extent to which COVID-19 indirectly shaped the latent risk dimensions identified in the PCA. However, the emergence of coherent risk clusters even under conditions of systemic stress underscores the importance of resilient maternal-care pathways capable of maintaining continuity of surveillance, risk assessment and support for vulnerable groups during large-scale public health disruptions.

### 4.5. Clinical and Public Health Implications

From a clinical standpoint, the findings support a shift from isolated, single-factor assessment of prematurity risk toward multidimensional evaluation that integrates maternal comorbidities, placental and obstetric characteristics, behavioural exposures and socio-demographic context. The cohort-specific PCA patterns underscore the need for tailored approaches to risk identification. In adolescent pregnancies, early recognition and management of hypertensive and other maternal complications remain critical, particularly because these factors tended to cluster alongside exogenous behaviours and adverse neonatal outcomes. Screening and counselling for substance use are especially relevant given their contribution to the latent structure of risk in this group.

In adult preterm pregnancies, the prominence of placental–obstetric and behavioural dimensions indicates that comprehensive evaluation of environmental context, delivery-related factors and lifestyle exposures is essential for timely intervention. For adult term pregnancies, behavioural and social determinants remained salient contributors to the latent risk structure, suggesting that preventive health promotion and targeted community programmes retain importance even when prematurity does not occur. From a public health perspective, the component structures identified here function as empirical summaries of how risks tend to cluster within distinct subpopulations, rather than as ready-to-use prediction models. These patterns may help inform resource allocation for adolescent reproductive health services, integrated antenatal–mental health–addiction programmes for women with complex risk profiles, and community-level interventions addressing smoking, alcohol use and other harmful exposures during pregnancy. Further work is required to convert these multidimensional patterns into validated risk scores and decision-support tools suitable for routine clinical practice.

### 4.6. Limitations and Future Research

This study has several limitations that must be acknowledged. The retrospective design and the reliance on data from a single tertiary maternity hospital in eastern Romania limit the generalisability of the findings to broader geographic and health-system contexts. Inclusion was restricted to women with complete data on the selected indicators, introducing the possibility of selection bias. As an observational study, the analysis does not allow causal inferences regarding the relationships between the identified latent dimensions and prematurity outcomes. The risk indicators were derived from routinely collected medical records, which may be affected by misclassification, incomplete reporting and variability in documentation practices. Important determinants of prematurity highlighted in the literature—such as psychosocial stress, intimate partner violence, detailed nutritional profiles or timing and intensity of substance use—were unavailable or insufficiently standardized for inclusion in the PCA. Also, the use of classical PCA on ordinal, clinically coded variables introduces methodological constraints. Although the ordered categories represent monotonic gradations along underlying clinical constructs and PCA is widely adopted in epidemiological research for exploratory dimensionality reduction, the method assumes continuous and linear relationships. Applying PCA to ordinal indicators may distort correlation structures or inflate shared variance. To address this, the analysis was explicitly framed as exploratory, and the interpretation focuses on patterns of co-occurrence rather than causal pathways. Future analyses will incorporate alternative approaches such as categorical PCA, polychoric PCA or multiple correspondence analysis to assess the robustness of these findings.

Next steps involve several planned directions. We will design prospective, multi-centre studies that integrate richer behavioural, psychosocial and biological measurements to refine the multidimensional risk structures identified here. We also plan to operationalise the PCA-derived components into explicit risk scores and evaluate their predictive accuracy and clinical utility in real-world settings. Additional work will test these latent structures across diverse health-system conditions, including periods of systemic stress such as pandemics. Through these efforts, we aim to contribute to more precise risk stratification and better targeted preventive and clinical interventions.

## 5. Conclusions

This study applied principal component analysis to a broad set of maternal, fetal, placental and behavioural indicators in adolescent preterm, adult preterm and adult term pregnancies from a tertiary maternity hospital in Eastern Europe. Across all three cohorts, three latent dimensions were identified, jointly explaining approximately two thirds of the total variance. Although the specific configurations of these components differed by cohort, the extracted structures consistently reflected coherent patterns of co-occurring risks rather than isolated determinants.

In adolescent preterm pregnancies, the latent structure indicated a close alignment between maternal complications, exogenous behaviours and prematurity-related neonatal outcomes. In adult preterm pregnancies, placental–obstetric indicators and behavioural factors formed distinct but interrelated dimensions, while fetal–neonatal markers contributed separately. Among term pregnancies, behavioural and socio-environmental indicators constituted the most prominent latent dimension, with fetal outcomes forming a separate component.

Taken together, these findings support a multidimensional and age-dependent conceptualisation of risk in pregnancy, emphasizing that prematurity emerges from combinations of interrelated clinical, behavioural and contextual factors. The PCA-derived dimensions presented here should be interpreted as exploratory representations of the underlying covariance structure, not as predictive or causal models. Future multicentre, prospective studies using richer psychosocial and behavioural data are needed to validate these patterns, develop operational risk scores and assess their applicability for enhancing risk stratification and early intervention in clinical practice.

## Figures and Tables

**Table 1:** Presentation of indicators.

No. Crt	Symbol	Name	Clinical Risk Stratification	Clinical Risk Type
1	MR_DM	Maternal risk associated with maternal comorbidities—Diabetes Mellitus	1. Low maternal risk (pregnant without diabetes); 2. High maternal risk (pregnant woman with diabetes).	Maternal
2	MR_HBP	Maternal risk associated with maternal comorbidities—Hypertension	1. Low maternal risk (pregnant without hypertension); 2. High maternal risk (pregnant woman with hypertension).	Maternal
3	PR_EnvUR	Placental risk associated with the degree of accessibility of health services in the residential setting	1. Low placental risk (services accessible in urban areas); 2. High placental risk (services less accessible in rural areas).	Placental
4	PR_PREMATURE	Level of placental risk by prematurity and fetal weight	1. Low placental risk—Normal delivery (fetal weight > 2500 g); 2. Medium placental risk—Prematurity I (Fetal weight < 2500 g); 3. High placental risk—Prematurity II (Fetal weight < 2000 g); 4. Very high placental risk—Prematurity III (Fetal weight < 1500 g); 5. Critical placental risk—Prematurity IV (Fetal weight < 1000 g).	Placental
5	PR_Studies	Placental risk associated with maternal lifestyle by level of education	1. Low placental risk (high level of knowledge: higher education); 2. Medium placental risk (medium knowledge level: 9–12 grades); 3. High placental risk (low knowledge level: 1–8 grades); 4. Very high placental risk (minimal knowledge level: no education); 5. Critical placental risk (level of knowledge unknown: educational level unspecified).	Placental
6	FR_BMALF	Fetal risk associated with possible fetal malformations at birth	1. Low fetal risk (Fetus born without malformations); 2. High fetal risk (Fetus born with malformations).	Fetal
7	FR_SPCD	Fetal risk associated with fetal health at hospital discharge	1. Low fetal risk (Fetus discharged with cured status); 2. Medium fetal risk (Fetus discharged with improved status); 3. High fetal risk (Fetus discharged with stationary status); 4. Very high fetal risk (Fetus discharged with worsened status); 5. Critical fetal risk (Fetus discharged with deceased status).	Fetal
8	FR_THRH	Fetal risk associated with clinical status requiring transfer to a higher-ranking hospital unit	1. Low fetal risk (no in-hospital transfer required); 2. High fetal risk (health status required in-hospital transfer).	Fetal
9	FR_TypeB	Fetal risk associated with type of birth	1. Low fetal risk (vaginal birth) 2. High fetal risk (cesarean delivery)	Fetal
10	ER_Alcohol	Exogenous risk associated with alcohol consumption during pregnancy	1. Low exogenous risk (non-drinking pregnant woman) 2. High exogenous risk (pregnant alcohol drinker)	Exogen
11	ER_Smoking	Exogenous risk associated with smoking during pregnancy	1. Low exogenous risk (pregnant non-smoker) 2. High exogenous risk (pregnant smoker)	Exogen
12	ER_Vices	Exogenous risk associated with cumulative vices during pregnancy	1. Low exogenous risk (pregnant non-users of dangerous substances: tobacco and alcohol) 2. High exogenous risk (pregnant user of dangerous substances: tobacco and alcohol)	Exogen

**Table 2:** Descriptive Statistics.

Variable	Mean “*E*” (Std. Deviation)	Mean “*P*” (Std. Deviation)	Mean “*M*” (Std. Deviation)
PR_EnvUR	1.537 (0.503)	1.459 (0.499)	1.47 (0.499)
PR_Studies	3.389 (0.596)	2 (0.758)	2.256 (0.902)
FR_TypeB	1.611 (0.492)	1.524 (0.501)	1.658 (0.474)
ER_Smoking	1.852 (0.359)	1.693 (0.462)	1.455 (0.498)
ER_Alcohol	1.315 (0.469)	1.199 (0.4)	1.277 (0.447)
ER_Vices	1.204 (0.407)	1.169 (0.375)	1.241 (0.428)
MR_DM	1.185 (0.392)	1.156 (0.363)	1.087 (0.282)
MR_HBP	1.444 (0.502)	1.212 (0.41)	1.094 (0.292)
PR_PREMATURE	2.407 (0.765)	2.29 (0.69)	1.000 (0.000)
FR_BMALF	1.056 (0.231)	1.026 (0.159)	1.005 (0.071)
FR_SPCD	1.833 (1.285)	1.537 (0.696)	1.336 (0.542)
FR_THRH	1.093 (0.293)	1.052 (0.222)	1.01 (0.099)

**Table 3:** Correlation analysis.

Variables	Lot	Correlation Sig. (1-Tailed)											
		PR_EnvUR	PR_Studies	FR_TypeB	ER_Smoking	ER_Alcohol	ER_Vices	MR_DM	MR_HBP	PR_PREMATURE	FR_BMALF	FR_SPCD	FR_THRH
PR_EnvUR	“*E*”	1 (0)	0.234 (0.044)	−0.741 (0)	−0.387 (0.002)	−0.65 (0)	−0.545 (0)	−0.513 (0)	−0.814 (0)	−0.285 (0.018)	−0.099 (0.238)	−0.267 (0.025)	−0.344 (0.005)
	“*P*”	1 (0)	−0.529 (0)	−0.896 (0)	−0.61 (0)	−0.416 (0)	−0.369 (0)	0.131 (0.023)	−0.457 (0)	0.319 (0)	0.068 (0.151)	0.577 (0)	0.058 (0.188)
	“*M*”	1 (0)	0.028 (0.055)	−0.744 (0)	−0.823 (0)	−0.58 (0)	−0.528 (0)	−0.245 (0)	−0.253 (0)	X	−0.042 (0.007)	0.51 (0)	−0.039 (0.011)
PR_Studies	“*E*”	0.234 (0.044)	1 (0)	−0.182 (0.094)	−0.432 (0.001)	0.026 (0.425)	0.134 (0.167)	0.17 (0.109)	−0.084 (0.273)	−0.313 (0.011)	−0.297 (0.015)	−0.283 (0.019)	−0.427 (0.001)
	“*P*”	−0.529 (0)	1 (0)	0.516 (0)	0.645 (0)	0.574 (0)	0.566 (0)	0.047 (0.237)	0.252 (0)	−0.283 (0)	0.108 (0.051)	−0.33 (0)	0.026 (0.348)
	“*M*”	0.028 (0.055)	1 (0)	−0.127 (0)	0.033 (0.03)	0.093 (0)	0.108 (0)	0.218 (0)	0.205 (0)	X	0.05 (0.002)	0.128 (0)	0.062 (0)
FR_TypeB	“*E*”	−0.741 (0)	−0.182 (0.094)	1 (0)	0.523 (0)	0.541 (0)	0.403 (0.001)	0.38 (0.002)	0.714 (0)	0.429 (0.001)	0.193 (0.08)	0.522 (0)	0.255 (0.031)
	“*P*”	−0.896 (0)	0.516 (0)	1 (0)	0.548 (0)	0.367 (0)	0.314 (0)	−0.116 (0.039)	0.431 (0)	−0.379 (0)	−0.117 (0.038)	−0.636 (0)	−0.05 (0.224)
	“*M*”	−0.744 (0)	−0.127 (0)	1 (0)	0.612 (0)	0.42 (0)	0.379 (0)	0.154 (0)	0.159 (0)	X	0.025 (0.075)	−0.717 (0)	−0.011 (0.262)
ER_Smoking	“*E*”	−0.387 (0.002)	−0.432 (0.001)	0.523 (0)	1 (0)	0.283 (0.019)	0.211 (0.063)	0.199 (0.075)	0.373 (0.003)	0.224 (0.052)	0.101 (0.233)	0.273 (0.023)	0.133 (0.168)
	“*P*”	−0.61 (0)	0.645 (0)	0.548 (0)	1 (0)	0.285 (0)	0.25 (0)	0.028 (0.339)	0.185 (0.002)	−0.591 (0)	−0.186 (0.002)	−0.458 (0)	−0.225 (0)
	“*M*”	−0.823 (0)	0.033 (0.03)	0.612 (0)	1 (0)	0.668 (0)	0.613 (0)	0.313 (0)	0.214 (0)	X	0.053 (0.001)	−0.394 (0)	0.055 (0.001)
ER_Alcohol	“*E*”	−0.65 (0)	0.026 (0.425)	0.541 (0)	0.283 (0.019)	1 (0)	0.746 (0)	0.293 (0.016)	0.758 (0)	0.372 (0.003)	0.184 (0.092)	0.339 (0.006)	0.196 (0.078)
	“*P*”	−0.416 (0)	0.574 (0)	0.367 (0)	0.285 (0)	1 (0)	0.904 (0)	0.144 (0.014)	0.457 (0)	−0.116 (0.04)	0.055 (0.203)	−0.323 (0)	−0.117 (0.038)
	“*M*”	−0.58 (0)	0.093 (0)	0.42 (0)	0.668 (0)	1 (0)	0.91 (0)	0.432 (0)	0.334 (0)	X	0.106 (0)	−0.226 (0)	0.087 (0)
ER_Vices	“*E*”	−0.545 (0)	0.134 (0.167)	0.403 (0.001)	0.211 (0.063)	0.746 (0)	1 (0)	0.469 (0)	0.565 (0)	0.031 (0.411)	0.279 (0.021)	−0.114 (0.205)	0.156 (0.13)
	“*P*”	−0.369 (0)	0.566 (0)	0.314 (0)	0.25 (0)	0.904 (0)	1 (0)	0.189 (0.002)	0.332 (0)	−0.089 (0.089)	0.072 (0.139)	−0.282 (0)	−0.105 (0.055)
	“*M*”	−0.528 (0)	0.108 (0)	0.379 (0)	0.613 (0)	0.91 (0)	1 (0)	0.461 (0)	0.339 (0)	X	0.117 (0)	−0.192 (0)	0.092 (0)
MR_DM	“*E*”	−0.513 (0)	0.17 (0.109)	0.38 (0.002)	0.199 (0.075)	0.293 (0.016)	0.469 (0)	1 (0)	0.533 (0)	−0.068 (0.314)	−0.116 (0.203)	−0.162 (0.121)	0.012 (0.465)
	“*P*”	0.131 (0.023)	0.047 (0.237)	−0.116 (0.039)	0.028 (0.339)	0.144 (0.014)	0.189 (0.002)	1 (0)	0.215 (0.001)	−0.164 (0.006)	−0.07 (0.144)	0.029 (0.332)	−0.101 (0.064)
	“*M*”	−0.245 (0)	0.218 (0)	0.154 (0)	0.313 (0)	0.432 (0)	0.461 (0)	1 (0)	0.62 (0)	X	0.156 (0)	0.015 (0.2)	0.151 (0)
MR_HBP	“*E*”	−0.814 (0)	−0.084 (0.273)	0.714 (0)	0.373 (0.003)	0.758 (0)	0.565 (0)	0.533 (0)	1 (0)	0.502 (0)	0.271 (0.024)	0.439 (0)	0.357 (0.004)
	“*P*”	−0.457 (0)	0.252 (0)	0.431 (0)	0.185 (0.002)	0.457 (0)	0.332 (0)	0.215 (0.001)	1 (0)	−0.065 (0.164)	−0.018 (0.392)	−0.249 (0)	−0.026 (0.347)
	“*M*”	−0.253 (0)	0.205 (0)	0.159 (0)	0.214 (0)	0.334 (0)	0.339 (0)	0.62 (0)	1 (0)	X	0.164 (0)	0.013 (0.232)	0.195 (0)
PR_PREMATURE	“*E*”	−0.285 (0.018)	−0.313 (0.011)	0.429 (0.001)	0.224 (0.052)	0.372 (0.003)	0.031 (0.411)	−0.068 (0.314)	0.502 (0)	1 (0)	0.616 (0)	0.876 (0)	0.587 (0)
	“*P*”	0.319 (0)	−0.283 (0)	−0.379 (0)	−0.591 (0)	−0.116 (0.04)	−0.089 (0.089)	−0.164 (0.006)	−0.065 (0.164)	1 (0)	0.445 (0)	0.489 (0)	0.355 (0)
FR_BMALF	“*E*”	−0.099 (0.238)	−0.297 (0.015)	0.193 (0.08)	0.101 (0.233)	0.184 (0.092)	0.279 (0.021)	−0.116 (0.203)	0.271 (0.024)	0.616 (0)	1 (0)	0.476 (0)	0.48 (0)
	“*P*”	0.068 (0.151)	0.108 (0.051)	−0.117 (0.038)	−0.186 (0.002)	0.055 (0.203)	0.072 (0.139)	−0.07 (0.144)	−0.018 (0.392)	0.445 (0)	1 (0)	0.344 (0)	0.207 (0.001)
	“*M*”	−0.042 (0.007)	0.05 (0.002)	0.025 (0.075)	0.053 (0.001)	0.106 (0)	0.117 (0)	0.156 (0)	0.164 (0)	X	1 (0)	0.149 (0)	0.291 (0)
FR_SPCD	“*E*”	−0.267 (0.025)	−0.283 (0.019)	0.522 (0)	0.273 (0.023)	0.339 (0.006)	−0.114 (0.205)	−0.162 (0.121)	0.439 (0)	0.876 (0)	0.476 (0)	1 (0)	0.293 (0.016)
	“*P*”	0.577 (0)	−0.33 (0)	−0.636 (0)	−0.458 (0)	−0.323 (0)	−0.282 (0)	0.029 (0.332)	−0.249 (0)	0.489 (0)	0.344 (0)	1 (0)	0.409 (0)
	“*M*”	0.51 (0)	0.128 (0)	−0.717 (0)	−0.394 (0)	−0.226 (0)	−0.192 (0)	0.015 (0.2)	0.013 (0.232)	X	0.149 (0)	1 (0)	0.328 (0)
FR_THRH	“*E*”	−0.344 (0.005)	−0.427 (0.001)	0.255 (0.031)	0.133 (0.168)	0.196 (0.078)	0.156 (0.13)	0.012 (0.465)	0.357 (0.004)	0.587 (0)	0.48 (0)	0.293 (0.016)	1 (0)
	“*P*”	0.058 (0.188)	0.026 (0.348)	−0.05 (0.224)	−0.225 (0)	−0.117 (0.038)	−0.105 (0.055)	−0.101 (0.064)	−0.026 (0.347)	0.355 (0)	0.207 (0.001)	0.409 (0)	1 (0)
	“*M*”	−0.039 (0.011)	0.062 (0)	−0.011 (0.262)	0.055 (0.001)	0.087 (0)	0.092 (0)	0.151 (0)	0.195 (0)	X	0.291 (0)	0.328 (0)	1 (0)

Note: X = not applicable; the indicator PR_PREMATURE is defined only for preterm births (Lots *E* and *P*), therefore the corresponding statistic for Lot *M* (term births) was not computed.

**Table 4:** KMO and Bartlett’s Test.

KMO and Bartlett’s Test		Lot = *E*	Lot = *P*	Lot = *M*
Kaiser-Meyer-Olkin (KMO) Measure of Sampling Adequacy.		0.609	0.718	0.749
Bartlett’s Test of Sphericity	Approx. Chi-Square	521.465	1723.008	21,126.927
	df	66	66	55
	Sig.	0.000	0.000	0.000

**Table 5:** Communalities.

Communalities	Initial	Extraction		
Variables		Lot = *E*	Lot = *P*	Lot = *M*
PR_EnvUR	1.000	0.807	0.852	0.808
PR_Studies	1.000	0.700	0.636	0.438
FR_TypeB	1.000	0.734	0.836	0.777
ER_Smoking	1.000	0.710	0.634	0.756
ER_Alcohol	1.000	0.750	0.845	0.738
ER_Vices	1.000	0.737	0.816	0.711
MR_DM	1.000	0.605	0.569	0.655
MR_HBP	1.000	0.867	0.367	0.550
PR_PREMATURE	1.000	0.883	0.701	X
FR_BMALF	1.000	0.649	0.525	0.555
FR_SPCD	1.000	0.719	0.664	0.716
FR_THRH	1.000	0.466	0.482	0.652

Note: X = not applicable; the indicator PR_PREMATURE is defined only for preterm births (Lots *E* and *P*), therefore the communalities for Lot *M* (term births) were not computed.

**Table 6:** Total Variance Explained.

**Total (% of Variance)**	**Initial Eigenvalues**					
**Component**	**Lot = *E***		**Lot = *P***		**Lot = *M***	
1	MR_HBP	4.92 (41%)	PR_EnvUR	4.534 (37.785%)	ER_Smoking	4.141 (37.642%)
2	PR_EnvUR	2.469 (20.577%)	FR_TypeB	1.964 (16.364%)	PR_EnvUR	2.079 (18.897%)
3	FR_TypeB	1.237 (10.306%)	ER_Smoking	1.428 (11.903%)	ER_Alcohol	1.135 (10.316%)
4	ER_Alcohol	1.012 (8.433%)	PR_Studies	0.98 (8.17%)	ER_Vices	0.89 (8.087%)
5	PR_PREMATURE	0.736 (6.129%)	FR_SPCD	0.923 (7.688%)	FR_TypeB	0.816 (7.42%)
6	FR_SPCD	0.57 (4.746%)	ER_Alcohol	0.725 (6.041%)	FR_SPCD	0.728 (6.62%)
7	ER_Vices	0.374 (3.118%)	ER_Vices	0.461 (3.843%)	MR_DM	0.432 (3.928%)
8	FR_THRH	0.33 (2.748%)	MR_HBP	0.337 (2.808%)	MR_HBP	0.368 (3.346%)
9	MR_DM	0.171 (1.424%)	FR_BMALF	0.292 (2.437%)	FR_BMALF	0.19 (1.727%)
10	FR_BMALF	0.102 (0.849%)	PR_PREMATURE	0.194 (1.619%)	FR_THRH	0.136 (1.236%)
11	ER_Smoking	0.053 (0.441%)	MR_DM	0.085 (0.705%)	PR_Studies	0.086 (0.78%)
12	PR_Studies	0.027 (0.229%)	FR_THRH	0.076 (0.635%)		0 (0%)
Cumulative%		100%		100%		100%
**Total (% of Variance)**	**Extraction Sums of Squared Loadings**			**Rotation Sums of Squared Loadings**		
**Component**	**Lot = *E***	**Lot = *P***	**Lot = *M***	**Lot = *E***	**Lot = *P***	**Lot = *M***
1	4.92 (41%)	4.534 (37.785%)	4.141 (37.642%)	3.83 (31.917%)	3.152 (26.267%)	3.695 (33.593%)
2	2.469 (20.577%)	1.964 (16.364%)	2.079 (18.897%)	3.035 (25.295%)	2.669 (22.243%)	2.183 (19.842%)
3	1.237 (10.306%)	1.428 (11.903%)	1.135 (10.316%)	1.76 (14.671%)	2.105 (17.542%)	1.476 (13.42%)
Cumulative%	71.883%	66.052%	66.855%	71.883%	66.052%	66.855%

**Table 7:** Component Matrix.

Variable	Lot/Component											
	E	1	2	3	P	1	2	3	M	1	2	3
MR_DM	9	0.399	−0.661	−0.089	11	0.082	0.078	0.746	7	0.526	0.551	−0.273
MR_HBP	1	0.896	−0.239	0.078	8	0.516	0.299	0.109	8	0.460	0.543	−0.210
PR_EnvUR	2	−0.819	0.324	0.175	1	−0.832	−0.012	0.399	2	−0.853	0.248	−0.136
PR_PREMATURE	5	0.677	0.610	0.228	10	−0.549	0.603	−0.189				
PR_Studies	12	−0.312	−0.528	0.569	4	0.734	0.297	−0.092	11	0.074	0.436	−0.492
FR_BMALF	10	0.470	0.518	0.400	9	−0.196	0.658	−0.233	9	0.125	0.436	0.590
FR_SPCD	6	0.605	0.590	0.076	5	−0.723	0.376	0.011	6	−0.503	0.675	0.082
FR_THRH	8	0.525	0.423	0.105	12	−0.265	0.453	−0.454	10	0.084	0.544	0.590
FR_TypeB	3	0.819	−0.121	−0.222	2	0.820	−0.061	−0.399	5	0.741	−0.440	0.185
ER_Alcohol	4	0.763	−0.319	0.255	6	0.684	0.528	0.314	3	0.840	0.168	−0.066
ER_Smoking	11	0.527	0.012	−0.657	3	0.745	−0.254	−0.120	1	0.856	−0.130	0.080
ER_Vices	7	0.580	−0.554	0.305	7	0.633	0.540	0.352	4	0.811	0.215	−0.088

Note: The colors highlight the intensity of direct and indirect correlations.

**Table 8:** Component Transformation Matrix.

Component	Lot = *E*			Lot = *P*			Lot = *E*		
	1	2	3	1	2	3	1	2	3
1	0.751	0.558	0.353	0.743	0.575	−0.342	0.894	0.437	0.097
2	−0.648	0.727	0.228	−0.136	0.630	0.764	−0.409	0.708	0.576
3	0.129	0.400	−0.907	−0.656	0.521	−0.546	0.183	−0.555	0.812

**Table 9:** Component Score Coefficient Matrix.

Variables	Component (Lot = E)			Component (Lot = P)			Component (Lot = M)		
	1	2	3	1	2	3	1	2	3
MR_DM	0.225	−0.178	0.033	−0.334	0.308	−0.261	−0.039	0.377	−0.030
MR_HBP	0.208	0.057	−0.015	0.014	0.201	0.036	−0.041	0.336	0.011
PR_EnvUR	−0.192	0.059	−0.157	−0.319	0.036	−0.095	−0.255	0.061	−0.048
PR_PREMATURE	−0.033	0.330	−0.062	−0.045	0.055	0.348	X	X	X
PR_Studies	0.150	−0.006	−0.489	0.142	0.155	0.096	−0.149	0.397	−0.229
FR_BMALF	−0.022	0.335	−0.212	0.030	0.101	0.360	0.036	−0.127	0.546
FR_SPCD	−0.055	0.267	0.043	−0.149	0.033	0.197	−0.228	0.137	0.234
FR_THRH	−0.020	0.218	0.000	0.134	−0.054	0.370	0.006	−0.094	0.575
FR_TypeB	0.133	−0.015	0.211	0.322	−0.061	0.067	0.276	−0.162	0.028
ER_Alcohol	0.227	0.075	−0.162	−0.069	0.371	0.034	0.138	0.178	0.019
ER_Smoking	0.009	−0.149	0.521	0.195	−0.031	−0.109	0.223	0.007	0.041
ER_Vices	0.266	0.001	−0.233	−0.095	0.382	0.028	0.118	0.202	0.016

Note: X = not applicable; the indicator PR_PREMATURE is defined only for preterm births (Lots *E* and *P*), hence the component score coefficient for Lot *M* (term births) was not computed.

## Data Availability

The original contributions presented in this study are included in the article. Further inquiries can be directed to the corresponding authors.

## Appendix A. The Full Cross-Tabulation of Categorical Risk Indicators by Cohort

**Variable**	**Risk**	**Test**	**Lot**			**Total**
			***E***	***M***	***P***	
PR_EnvUR	1. Low placental risk	Count	25	1776	125	1926
		% within PR_EnvUR	1.3%	92.2%	6.5%	100.0%
	2. High placental risk	Count	29	1578	106	1713
		% within PR_EnvUR	1.7%	92.1%	6.2%	100.0%
Total		Count	54	3354	231	3639
		% within PR_EnvUR	1.5%	92.2%	6.3%	100.0%
PR_Studies	1. Low placental risk	Count	0	710	60	770
		% within PR_Studies	0.0%	92.2%	7.8%	100.0%
	2. Medium placental risk	Count	2	1378	115	1495
		% within PR_Studies	0.1%	92.2%	7.7%	100.0%
	3. High placental risk	Count	30	1000	54	1084
		% within PR_Studies	2.8%	92.3%	5.0%	100.0%
	4. Very high placental risk	Count	21	231	0	252
		% within PR_Studies	8.3%	91.7%	0.0%	100.0%
	5. Critical placental risk	Count	1	35	2	38
		% within PR_Studies	2.6%	92.1%	5.3%	100.0%
Total		Count	54	3354	231	3639
		% within PR_Studies	1.5%	92.2%	6.3%	100.0%
FR_TypeB	1. Low fetal risk	Count	21	1146	110	1277
		% within FR_TypeB	1.6%	89.7%	8.6%	100.0%
	2. High fetal risk	Count	33	2208	121	2362
		% within FR_TypeB	1.4%	93.5%	5.1%	100.0%
Total		Count	54	3354	231	3639
		% within FR_TypeB	1.5%	92.2%	6.3%	100.0%
ER_Smoking	1. Low exogenous risk	Count	8	1828	71	1907
		% within ER_Smoking	0.4%	95.9%	3.7%	100.0%
	2. High exogenous risk	Count	46	1526	160	1732
		% within ER_Smoking	2.7%	88.1%	9.2%	100.0%
Total		Count	54	3354	231	3639
		% within ER_Smoking	1.5%	92.2%	6.3%	100.0%
ER_Alcohol	1. Low exogenous risk	Count	37	2426	185	2648
		% within ER_Alcohol	1.4%	91.6%	7.0%	100.0%
	2. High exogenous risk	Count	17	928	46	991
		% within ER_Alcohol	1.7%	93.6%	4.6%	100.0%
Total		Count	54	3354	231	3639
		% within ER_Alcohol	1.5%	92.2%	6.3%	100.0%
ER_Vices	1. Low exogenous risk	Count	43	2547	192	2782
		% within ER_Vices	1.5%	91.6%	6.9%	100.0%
	2. High exogenous risk	Count	11	807	39	857
		% within ER_Vices	1.3%	94.2%	4.6%	100.0%
Total		Count	54	3354	231	3639
		% within ER_Vices	1.5%	92.2%	6.3%	100.0%
MR_DM	1. Low maternal risk	Count	44	3061	195	3300
		% within MR_DM	1.3%	92.8%	5.9%	100.0%
	2. High maternal risk	Count	10	293	36	339
		% within MR_DM	2.9%	86.4%	10.6%	100.0%
Total		Count	54	3354	231	3639
		% within MR_DM	1.5%	92.2%	6.3%	100.0%
MR_HBP	1. Low maternal risk	Count	30	3038	182	3250
		% within MR_HBP	0.9%	93.5%	5.6%	100.0%
	2. High maternal risk	Count	24	316	49	389
		% within MR_HBP	6.2%	81.2%	12.6%	100.0%
Total		Count	54	3354	231	3639
		% within MR_HBP	1.5%	92.2%	6.3%	100.0%
PR_PREMATURE	1. Low placental risk	Count	0	3354	0	3354
		% within PR_PREMATURE	0.0%	100.0%	0.0%	100.0%
	2. Medium placental risk	Count	40	0	187	227
		% within PR_PREMATURE	17.6%	0.0%	82.4%	100.0%
	3. High placental risk	Count	7	0	29	36
		% within PR_PREMATURE	19.4%	0.0%	80.6%	100.0%
	4. Very high placental risk	Count	6	0	7	13
		% within PR_PREMATURE	46.2%	0.0%	53.8%	100.0%
	5. Critical placental risk	Count	1	0	8	9
		% within PR_PREMATURE	11.1%	0.0%	88.9%	100.0%
Total		Count	54	3354	231	3639
		% within PR_PREMATURE	1.5%	92.2%	6.3%	100.0%
FR_BMALF	1. Low fetal risk	Count	51	3337	225	3613
		% within FR_BMALF	1.4%	92.4%	6.2%	100.0%
	2. High fetal risk	Count	3	17	6	26
		% within FR_BMALF	11.5%	65.4%	23.1%	100.0%
Total		Count	54	3354	231	3639
		% within FR_BMALF	1.5%	92.2%	6.3%	100.0%
FR_SPCD	1. Low fetal risk	Count	32	2307	124	2463
		% within FR_SPCD	1.3%	93.7%	5.0%	100.0%
	2. Medium fetal risk	Count	12	998	98	1108
		% within FR_SPCD	1.1%	90.1%	8.8%	100.0%
	3. High fetal risk	Count	1	23	3	27
		% within FR_SPCD	3.7%	85.2%	11.1%	100.0%
	4. Very high fetal risk	Count	5	20	4	29
		% within FR_SPCD	17.2%	69.0%	13.8%	100.0%
	5. Critical fetal risk	Count	4	6	2	12
		% within FR_SPCD	33.3%	50.0%	16.7%	100.0%
Total		Count	54	3354	231	3639
		% within FR_SPCD	1.5%	92.2%	6.3%	100.0%
FR_THRH	1. Low fetal risk	Count	49	3321	219	3589
		% within FR_THRH	1.4%	92.5%	6.1%	100.0%
	2. High fetal risk	Count	5	33	12	50
		% within FR_THRH	10.0%	66.0%	24.0%	100.0%
Total		Count	54	3354	231	3639
		% within FR_THRH	1.5%	92.2%	6.3%	100.0%
